# Sox enters the picture

**DOI:** 10.7554/eLife.41136

**Published:** 2018-10-01

**Authors:** Felix Kaufholz, Natascha Turetzek

**Affiliations:** 1 Department of Evolutionary Developmental GeneticsGeorg-August-Universität GöttingenGoettingenGermany; 2 Evolutionary Ecology, Department Biology IILudwig-Maximilians Universität MünchenMartinsriedGermany

**Keywords:** parasteatoda tepidariorum, spider, evolution, development, segmentation, evolution of segmentation, Other

## Abstract

The discovery of a gene that regulates two segmentation mechanisms in spider embryos is fueling the ongoing debate about the evolution of this crucial developmental process.

**Related research article** Paese CLB, Schoenauer A, Leite DJ, Russell S, McGregor AP. 2018. A SoxB gene acts as an anterior gap gene and regulates posterior segment addition in a spider. *eLife* **7**:e37567. doi: 10.7554/eLife.37567

It is usually assumed that humans have little in common with arthropods, such as insects and spiders, or annelids such as worms. However, the body plans of vertebrates, arthropods and annelids share a striking feature: the body is subdivided into distinct segments (pink dot, Figure 1A), and scientists have been asking "is segmentation evolutionarily conserved?" for more than a century.

**Figure 1.**
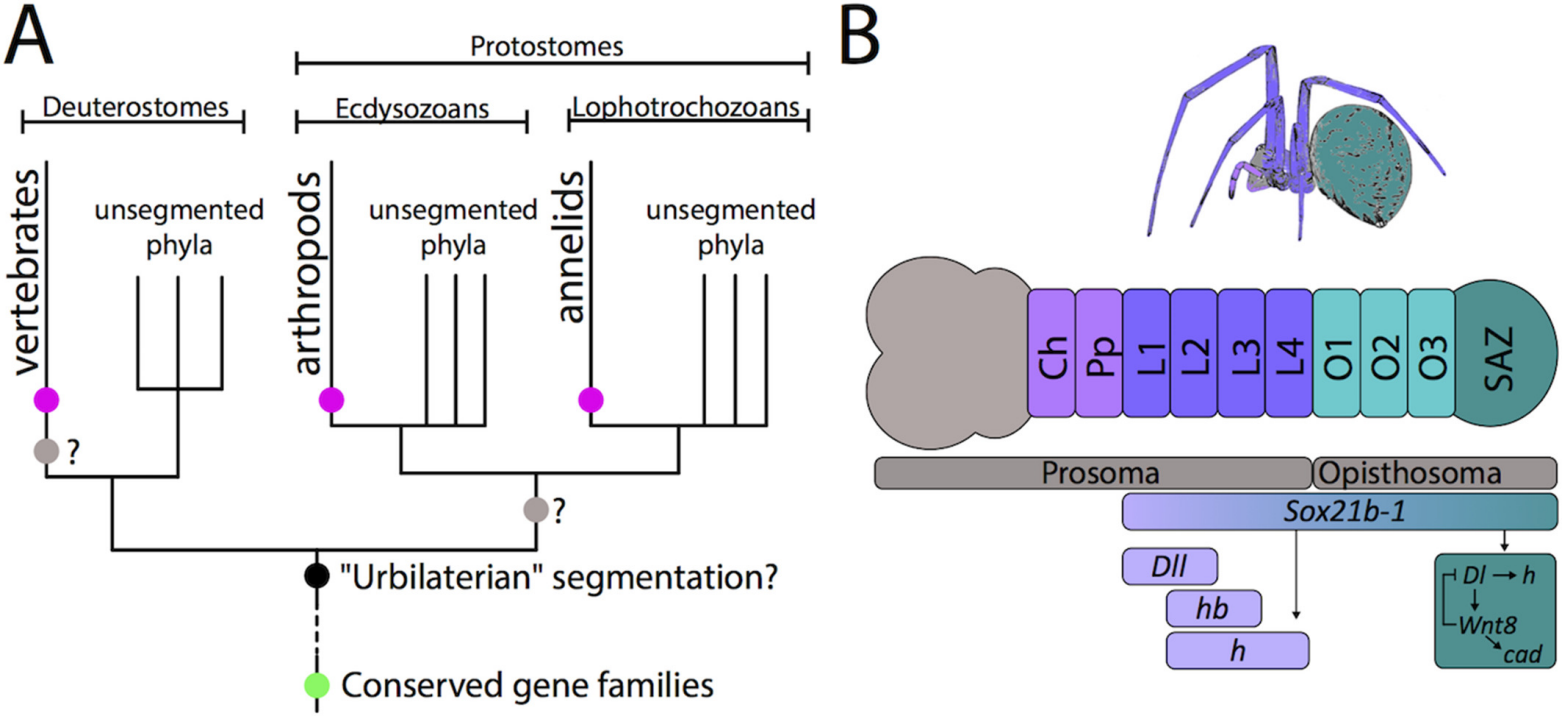
The evolution of segmentation. (**A**) Schematic representation of the phylogenetic relationships of bilaterians (adapted from Peel and Akam, 2003). Although vertebrates, arthropods, and annelids all have a segmented anterior-posterior body axis (pink dots), they are all closely related to phyla that are not segmented. Some conserved gene families (including Sox and Wnt) were already present before the emergence of the bilaterians (green dot) and are involved in many important developmental processes: some are also known to be involved in segmentation in various animals. However, the evolution of segmentation is still under debate. It could have evolved once in the 'urbilateria' (black dot), the last common ancestor of all bilaterians, or twice – at the base of the vertebrates and independently at the base of the protostomes (grey dots). Both of these scenarios would suggest a subsequent loss of segmentation in all closely related unsegmented phyla. The third scenario is that segmentation independently evolved three times – at the base of the vertebrates, arthropods, and annelids (pink dots). (**B**) Schematic representation of the germband and related adult tissues of the spider *Parasteatoda tepidariorum* (adapted from Paese et al., 2018). The body of a spider embryo is comprised of the prosoma (including the head (grey) and thorax (violet and indigo) and the opisthsoma (green). The formation of the leg-bearing prosomal segments (L1–L4) depends on the gap-like functions of genes such as *Distal-less (Dll), hairy (h), hunchback (hb)* and, as now shown by Paese et al., *Sox21b-1*. The opisthosomal segments are added sequentially by a segment addition zone (SAZ, dark green) controlled by a complex gene regulatory network (dark green box), probably induced by *Sox21b-1,* that contains *caudal (cad)* and components of the Wnt (*Wnt8*) and Notch (*Delta*) signaling pathways. Ch: Cheliceral segment. Pp: Pedipalpal segment. *Dl: Delta*.

In vertebrate embryos, segments are added progressively along the body axis from the anterior (head) to the posterior (tail). This process involves oscillating patterns of gene expression in the posterior of the organism, including widely conserved genes that code for proteins like *caudal* and components of the well-known Notch and Wnt signaling pathways.

In the vinegar fly *Drosophila melanogaster*, on the other hand, the segments are formed almost simultaneously early during embryonic development following a complex cascade of gene interactions, in a process known as long-germband segmentation. Maternally-provided factors, such as *caudal,* induce gap genes, which subsequently control the expression of pair-rule genes and, finally, segment-polarity genes (including a Wnt homolog). The end result is the formation of a molecular pre-pattern for segmentation.

For many years these morphological and genetic differences were considered as strong evidence that segmentation does not have a common origin. However, other arthropods employ an approach called short-germband segmentation that is, at least morphologically, more similar to the approach taken by vertebrates. In short-germband segmentation a small number of anterior segments are defined more or less simultaneously by gap (or gap-like) genes, while the remaining segments are added sequentially to the posterior by the 'segment addition zone' (Figure 1B).

A revolution in the field of evolutionary developmental biology was triggered in 2003 when researchers demonstrated that the segment addition zone in spider embryos depends on Notch signaling, as it does in vertebrates (Stollewerk et al., 2003). Additional comparative studies in spiders, insects and other arthropods revealed that short-germband segmentation with a segment addition zone depending on *caudal*, Notch and Wnt signaling, probably represents the ancestral mode of patterning in arthropods, whereas the genetic cascade in long-germband insects represents a derived state.

Recent studies, however, revealed even deeper similarities than previously thought among the complex genetic mechanisms underlying long- and short-germband segmentation. These included a more detailed analysis of gene expression dynamics and regulation in *D. melanogaster* and the flour beetle *Tribolium castaneum* (Clark and Akam, 2016; Clark and Peel, 2018; Zhu et al., 2017) and the discovery of oscillatory gene expression dynamics in long-germband insects (Verd et al., 2018).

Now, in eLife, Alistair McGregor and co-workers at Oxford Brookes University and Cambridge University – including Christian Paese of Oxford Brookes as first author – report another piece of evidence for this deeper level of conservation of segmentation by showing that a gene called *Sox21b-1* is involved in segmentation in the spider *Parasteatoda tepidariorum* (Paese et al., 2018). *P. tepidariorum* has emerged as a model system in which to study the influences of whole genome duplications on development in arthropods (Schwager et al., 2017).

*Sox21b-1* belongs to the B group of the Sox family of transcription factors, which are present in all metazoan species. In arthropods the SoxB group is comprised of *SoxNeuro*, *Sox21a*, *Sox21b* and *Dichaete*. Although the evolution of arthropod SoxB genes is not fully resolved yet, *Sox21b* and *Dichaete* are closely related and probably arose by duplication in the last common ancestor of the arthropods, while in onychophorans (velvet worms), the sister group to arthropods, only one *Dichaete*/*Sox21b* class gene seems to be present (Janssen et al., 2018).

In the current study Paese et al. demonstrate that *Sox21b* underwent a second round of duplication in spiders, probably during a whole genome duplication event (Schwager et al., 2017), giving rise to the two paralogs: *Sox21b-1* and *Sox21b-2.* Using RNA interference Paese et al. found that a knockdown of the *Sox21b-1* paralog resulted in both the loss of leg-bearing prosomal segments and opisthosomal segments, including loss of the entire segment addition zone (Figure 1B). In severe cases, even proper formation of the germband itself was disrupted. The researchers also explored where *Sox21b-1* is located in the genetic cascade that controls segmentation in the spider (Schönauer et al., 2016). They found that in addition to acting as a gap-like gene in the prosoma, *Sox21b-1* also regulates the expression of another gap-like gene and of many genes (including *caudal* and components of the Wnt and Notch signaling pathways) that are required to set up the segment addition zone in the opisthosoma.

These findings are striking for many reasons. Sox genes are known to be involved in segmentation, and to interact with Wnt genes, in both insects and vertebrates (Clark and Peel, 2018; Javali et al., 2017; Mukherjee et al., 2000; Russell et al., 1996). Moreover, both the Sox and Wnt gene families belong to the ancient gene repertoire of all bilaterians and have experienced multiple duplication events during evolution. *Dichaete* is known to control the expression of pair-rule genes in *D*. *melanogaster* (Russell et al., 1996), but relatively little is known about *Sox21b*. More recent studies in *T*. *castaneum* demonstrated that both *Dichaete* and *Sox21b* are expressed in the segment addition zone (Clark and Peel, 2018; Janssen et al., 2018), which suggests that the role of SoxB genes in segmentation is conserved for long- and short- germband species.

Expression ofSoxB and Wnt genes has also been found in the most posterior part of velvet worm embryos (Hogvall et al., 2014; Janssen et al., 2018). Together with the latest results from the spider, this provides further support for a conserved genetic basis (involving homologs of SoxB and Wnt genes) for the different segmentation modes of arthropods.

The astonishing results reported by Paese et al. highlight once again how far we are from a complete understanding of segmentation. It also underlines the need for further comparative studies in various species, focusing on conserved gene families, especially after duplication events, to determine if segmentation evolved anciently or independently in the three segmented bilaterian lineages (pink dot, Figure 1A). Techniques like RNA interference, CRISPR/Cas9 and new sequencing methods, together with an increasing number of genomes and transcriptomes available for emerging model organisms, will hopefully help to answer this question.
